# Impact of Atherosclerosis- and Diabetes-Related Dicarbonyls on Vascular Endothelial Permeability: A Comparative Assessment

**DOI:** 10.1155/2017/1625130

**Published:** 2017-10-02

**Authors:** Mikhail V. Samsonov, Asker Y. Khapchaev, Alexander V. Vorotnikov, Tatyana N. Vlasik, Elena V. Yanushevskaya, Maria V. Sidorova, Evgeniy E. Efremov, Vadim Z. Lankin, Vladimir P. Shirinsky

**Affiliations:** ^1^Russian Cardiology Research and Production Complex, Ministry of Healthcare of Russian Federation, 3rd Cherepkovskaya St. 15a, Moscow 121552, Russia; ^2^Faculty of Fundamental Medicine, Lomonosov Moscow State University, Lomonosovsky Ave., 27, Moscow 119192, Russia

## Abstract

**Background:**

Malondialdehyde (MDA), glyoxal (GO), and methylglyoxal (MGO) levels increase in atherosclerosis and diabetes patients. Recent reports demonstrate that GO and MGO cause vascular endothelial barrier dysfunction whereas no evidence is available for MDA.

**Methods:**

To compare the effects of MDA, GO, or MGO on endothelial permeability, we used human EA.hy926 endothelial cells as a standard model. To study cortical cytoplasm motility and cytoskeletal organization in endothelial cells, we utilized time-lapse microscopy and fluorescent microscopy. To compare dicarbonyl-modified protein band profiles in these cells, we applied Western blotting with antibodies against MDA- or MGO-labelled proteins.

**Results:**

MDA (150–250 *μ*M) irreversibly suppressed the endothelial cell barrier, reduced lamellipodial activity, and prevented intercellular contact formation. The motile deficiency of MDA-challenged cells was accompanied by alterations in microtubule and microfilament organization. These detrimental effects were not observed after GO or MGO (250 *μ*M) administration regardless of confirmed modification of cellular proteins by MGO.

**Conclusions:**

Our comparative study demonstrates that MDA is more damaging to the endothelial barrier than GO or MGO. Considering that MDA endogenous levels exceed those of GO or MGO and tend to increase further during lipoperoxidation, it appears important to reduce oxidative stress and, in particular, MDA levels in order to prevent sustained vascular hyperpermeability in atherosclerosis and diabetes patients.

## 1. Introduction

Patients with atherosclerosis and type 2 diabetes often demonstrate elevated endogenous dicarbonyls including malondialdehyde (MDA), glyoxal (GO), and methylglyoxal (MGO) [1]. The level of MDA serves as a clinically validated biomarker of oxidative stress [2, 3]. The major endogenous source of GO and MGO is believed to be high blood glucose and other carbohydrates [4, 5] whereas MDA is produced mainly from polyunsaturated fatty acids (PUFA) during free radical lipoperoxidation. This condition accompanies oxidative stress and is typical to both atherosclerosis and diabetes [2, 6]. The aldehyde/carbonyl groups of GO, MGO, and MDA may react with proteins, nucleic acids, and other biopolymers, which critically contributes to the pathological effects of these reactive dicarbonyl species. In particular, these substances irreversibly modify proteins in vascular cells leading to micro- and macrovasculopathies that manifest in accelerated atherosclerotic plaque progression, augmented endothelial permeability, and consecutive deterioration of underlying tissues [5]. In addition, natural dicarbonyls cause modification of apoprotein B-100 in LDL particles, which contributes to their enhanced uptake by vessel wall cells during atherosclerotic lesion development [2]. The organism possesses enzymatic systems to detoxify these aldehyde species; however, under conditions of severe oxidative stress, these enzymes would be partially inactivated [7].

The effects of GO and MGO on endothelial permeability were addressed in a number of studies [8–11]. Surprisingly, the effects of MDA received much less attention. Based on chemical differences between these compounds, relative severity of their action on the endothelial barrier is expected to vary, but experimental evidence addressing this issue is limited.

To fill this gap in knowledge, we used a standard human EA.hy926 endothelial cell line as an in vitro model of endothelial monolayer to compare the effects of MDA, MGO, or GO on permeability, cytoskeletal organization, and motile behavior of these cells. We report that MDA potently impairs the endothelial barrier at concentrations approaching those in disease, whereas GO or MGO produces no marked effects on endothelial permeability regardless of the confirmed endothelial protein modifications.

## 2. Materials and Methods

### 2.1. Reagents

General reagents, gelatin, anti-*β*-tubulin mouse monoclonal antibody (moAb) and peroxidase-conjugated secondary antibodies, carnosine, lysine, N-acetyl cysteine (NAC), GO, MGO, and glutaraldehyde (GA) were purchased from Sigma. The actual concentration of GO and MGO in stock solutions was established using a standard protocol of dicarbonyl derivatization with *o*-phenylenediamine followed by quantification of the resulting quinoxaline/2-methylquinoxaline with high-performance liquid chromatography [12]. According to analysis, the content of GO and MGO in stock solutions was at least 90% of that stated by the vendor. MDA was obtained by acid hydrolysis of 1,1,4,4-tetrahydroxypropane according to the published protocol [13]. Other reagents were obtained from the following vendors. Alexa Fluor 546-conjugated goat anti-mouse IgG, Alexa Fluor 594 phalloidin, and 4′,6-diamidino-2-phenylindole (DAPI) were from Invitrogen. Hank's balanced salt solution (HBSS), DMEM, and EGM-2mv endothelial growth medium were from Lonza. Fetal bovine serum (FBS), trypsin-EDTA solution, and antibiotic solution were from HyClone. Active human *α*-thrombin was from Enzyme Research Laboratories. Cell culture plastic ware and ThinCerts, 0.4 *μ*m porous membrane inserts for diffusion chambers, were from Greiner Bio-One. *μ*-Slide I^0^^.4^ Luer and 8W1E arrays for the ECIS-z electric cell impedance measuring system (Applied Biophysics, USA) were from http://www.ibidi.de, Germany. Clarity ECL Western blotting substrate and precast Criterion gradient gels were from Bio-Rad. PVDF Hybond-P membrane was from Amersham. EA.hy926 human endothelial cell line [14] was initially obtained from ATСС (CRL 2922, Manassas, Virginia, USA) and propagated in the laboratory with reproducible performance in permeability experiments [15, 16].

### 2.2. Mouse Monoclonal Antibodies to MDA- or MGO-Modified Proteins

The monoclonal antibodies were produced at the Cardiology Research and Production Complex using standard procedures for mouse immunization and hybridoma generation. In brief, BALB/c mice were repeatedly immunized using low-density lipoproteins (LDL) isolated from blood plasma of healthy donors and modified in vitro by MDA or MGO [17, 18]. Antibodies (IgG) were isolated from ascitic fluids by ammonium sulfate precipitation followed by ion-exchange chromatography. Subsequent testing of moAb to MDA-modified LDL (clone 3G4) and MGO-modified LDL (clone 6D8) using ELISA and in vitro-modified protein standards (LDL, human serum albumin, human IgG, etc.) revealed that the obtained moAb recognized MDA-labelled (clone 3G4) or MGO-labelled (clone 6D8) proteins and did not recognize unmodified proteins. Results obtained in the present work further confirm these findings. There was no moAb reactivity in immunoblotting with proteins of untreated EA.hy926 cells as opposed to MDA- or MGO-treated cells (see Section 3.4). Additionally, we observed no cross-reactivity in immunoblotting of anti-MDA moAb with the proteins of EA.hy926 cells treated with MGO or GO. Neither anti-MGO moAb recognized proteins of EA.hy926 cells treated with MDA or GO (see Supplement Figure 1S in Supplementary Material available online at https://doi.org/10.1155/2017/1625130). Thus, we produced moAb that specifically recognizes MDA- or MGO-modified proteins in ELISA and immunoblotting applications.

### 2.3. Endothelial Cell Culture and Cell Manipulation

EA.hy926 cells were cultured on 0.2% gelatin-coated 100 mm Petri dishes in DMEM with normal (1 g/L) glucose and without pyruvate, supplemented with 200 *μ*M L-glutamine, 100 U/mL penicillin, 100 *μ*g/mL streptomycin, and 10% fetal calf serum at 37°С and 5% CO_2_/95% air atmosphere. The growth medium was changed every other day. Cells were split to 1 : 3 using 0.25% trypsin-EDTA. Prior to experiments, cells were transferred in EGM-2mv for 1.5–2 days to augment their barrier capacity (Samsonov M., unpublished observations).

### 2.4. Transendothelial Electric Resistance Experiments

EA.hy926 cells were seeded in 300 *μ*L DMEM containing 10% FBS at 10^5^ cells/well in individual wells of 8W1E electrode arrays covered with 0.2% gelatin and stabilized according to the manufacturer's instructions. After cell attachment and spreading, DMEM growth medium was replaced with 300 *μ*L of EGM-2mv growth medium and cells were further cultured for 1.5 days. The growth medium was replaced with 300 *μ*L HBSS ahead of experiment, and the total electric impedance of the quiescent confluent cell monolayer was monitored for 1 hour using an ECIS-z system (Applied Biophysics, USA). Next, carbonyls (100 to 250 *μ*M) or 100 nM thrombin was added to the wells, and the impedance was monitored for up to 19 hours.

### 2.5. FITC-Dextran Diffusion Experiments

ThinCerts were coated with 0.2% gelatin; 10^5^ EA.hy926 cells were seeded onto porous membranes in order to produce a confluent monolayer upon cell spreading. EGM-2mv growth medium was added to the top (200 *μ*L) and the bottom (800 *μ*L) compartments of the diffusion chamber, and cells were left for 1.5 days in a CO_2_ incubator. Next, growth medium in both compartments was replaced with HBSS supplemented with 250 *μ*M MDA, MGO, or GO. After 4 hours of cell incubation with aldehydes, 5 *μ*L of 10 mg/mL FITC-dextran was added to the top compartment and cells were incubated for additional 15 hours in a CO_2_ incubator. At the end of the experiment, 10 *μ*L medium was taken from the lower compartment of the diffusion chamber and diluted in 100 *μ*L HBSS. Fluorescence intensity of FITC-dextran was measured using the Victor X3 plate reader (Perkin Elmer, USA) at *λ*_ex_ = 495 nm and *λ*_em_ = 535 nm.

### 2.6. Time-Lapse Microscopy of Endothelial Cells and Analysis of Lamellipodial Activity

EA.hy926 cells were seeded in 0.2% gelatin-covered *μ*-Slide I^0.4^ at 0.5 × 10^5^ cells per slide and incubated in EGM-2mv growth medium for 1.5 days until subconfluence. Growth medium was changed every 12 hours. Cells were washed with HBSS and challenged with 250 *μ*M dicarbonyl solution in HBSS for 4 hours in a CO_2_ incubator. Then, the *μ*-Slide was placed under the microscope without washing out dicarbonyls. Time-lapse recording of live EA.hy926 cells was performed using a motorized Axiovert 200M inverted microscope equipped with an on-stage thermostat (37°C) and an AxioCam HR_m_ CCD camera (Zeiss, Germany). Movies were recorded using a 40x objective in the phase-contrast mode taking one frame per 20 sec for 1 hour. Images were collected and processed using the AxioVision 4.8.2 software (Zeiss, Germany), the ImageJ freeware (NIH, Bethesda, USA), and Adobe Photoshop CS6 (Adobe Corp., USA).

Lamellipodial activity of individual cells was quantified from these movies using the ImageJ freeware. The perimeter of a lamellipodium that expanded/retracted from its initial position during 100 sec period was traced using a freehand selection tool. It was then converted in the number of pixels contained within the outlined perimeter using the “measure” function of ImageJ. Measurements from 10–12 cells were averaged and expressed as the speed of lamellipodial area expansion/retraction in pixels/min.

### 2.7. Fluorescent Microscopy of Endothelial Cells

EA.hy926 cells were seeded on glass coverslips precoated with 0.2% gelatin and cultured in EGM2-mv growth medium to subconfluency. Then, the cells were washed once with HBSS and incubated for 5 hours in HBSS (control) or HBSS supplemented with 250 *μ*M GO, MGO, or MDA in a CO_2_ incubator. Microtubules and microfilaments were visualized in the cells as described previously [19]. Cell nuclei were stained with 1 *μ*g/mL DAPI for 10 min. Fluorescent images were acquired, processed, and assembled using the equipment and software described in time-lapse microscopy subsection.

### 2.8. Electrophoresis and Western Blotting of Endothelial Cell Lysates

EA.hy926 cells were seeded onto 35 mm Petri dishes coated with 0.2% gelatin in DMEM containing 10% FBS. The medium was exchanged to EGM-2mv growth medium two days before the experiment and to HBSS immediately prior to the experiment. MDA, MGO, or GO was added to the cells in HBSS at a final concentration of 250 *μ*Μ and incubated for 5 hours. The cells were washed twice with an ice-cold PBS and lysed in 2^x^ SDS sample buffer containing the Roche Complete Mini EDTA-free protease inhibitor cocktail (Sigma-Aldrich, USA). The lysates were passed 5 times through a 30-gauge syringe needle to disrupt DNA, boiled for 5 min, and clarified by centrifugation at 16,000*g* at 4°C for 10 min. Western blotting was performed according to standard protocols [20, 21]. The protein bands were detected by chemiluminescence using Clarity ECL reagents (Bio-Rad, USA) and the Fusion-SL 3500WL visualization system (Vilber Lourmat, France).

### 2.9. Statistics

The data were analyzed using Student's *t*-test and presented as mean ± SD. *p* < 0.05 was considered statistically significant. Experiments were carried out in triplicates or quadruplicates and repeated at least three times.

## 3. Results

### 3.1. Differential Effects of MDA versus GO/MGO on Endothelial Permeability

**Figure 1:**
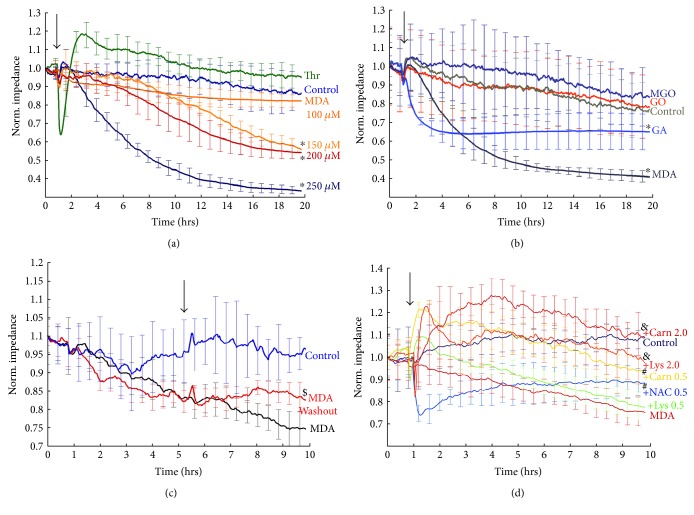
Effects of dicarbonyls and thrombin on the transendothelial electric resistance of the EA.hy926 cell monolayer. (a) Time course of dose-dependent TER changes produced by the indicated MDA concentrations and 100 nM thrombin (Thr); (b) time course of TER change produced by 250 *μ*M MDA, GO, MGO, or glutaraldehyde (GA); (c) effects of MDA washout on the time course of TER decline produced by 200 *μ*M MDA; (d) time course of TER change produced by 200 *μ*M MDA in the presence of 0.5 mM NAC, lysine, or carnosine and in the presence of 2 mM lysine or carnosine. Control, cells treated with HBSS without dicarbonyls added. The arrow indicates the addition of either dicarbonyl (a–d) and thrombin (a) or MDA washout (c). TER is expressed as the normalized impedance. Data from three independent experiments were pooled. ^∗^*p* < 0.05 versus control, MGO, GO, or thrombin for the marked conditions at 20 hours (*n* = 3–9). ^$^*p* < 0.05 versus both control and MDA at 10 hours (*n* = 3). ^#^*p* < 0.05 versus both control and MDA at 10 hours (*n* = 4). ^&^*p* < 0.05 versus MDA at 10 hours (*n* = 4).

At concentrations of 150–250 *μ*M, MDA produced a dose-dependent decrease in the total electric impedance of the EA.hy926 cell monolayer measured by the transendothelial electric resistance (TER) assay (Figure 1(a)). This indicates that MDA increases endothelial permeability. However, the loss of the barrier function induced by MDA developed over hours, which contrasts the fast increase in permeability induced by thrombin. By amplitude, the MDA-induced decrease in TER was similar to that elicited by thrombin. Because the effect of 250 *μ*M MDA reached saturation during the observation period, this concentration was chosen to compare the effects of MDA to those of GO or MGO on TER of the EA.hy926 cell monolayer (Figure 1(b)). In contrast to MDA, neither GO nor MGO decreased TER at concentrations of 250 *μ*M as compared to vehicle-treated time controls.

We also used artificial dicarbonyl, glutaraldehyde (GA, 250 *μ*M), which acted similar to MDA and decreased TER of EA.hy926 cells. The effect of glutaraldehyde developed faster and saturated in 3.5–4 hours.

In separate experiments, we checked whether the effect of MDA was irreversible. After a 5-hour exposure, MDA was washed out. This prevented the further decline in TER; however, there was no recovery toward the original resistance values within the next 5 hours of experiment (Figure 1(c)), or later when HBSS was replaced with growth medium in MDA-treated cells (data not shown). Thus, MDA exerted a long-lasting effect on TER of EA.hy926 endothelial cells, and this effect could be considered irreversible in contrast to the effect of thrombin that was over within an hour (Figure 1(a)).

Finally, we used the TER assay to establish whether amino group-containing compounds or antioxidants could neutralize the negative effect of MDA on the endothelial barrier. For this purpose, we used a free amino acid lysine, dipeptide carnosine (*β*-alanyl-L-histidine), and antioxidant N-acetyl cysteine (NAC). When these substances were added at 0.5 mM in HBSS along with 200 *μ*M MDA, only the carnosine readily prevented the decrease in TER produced by MDA (Figure 1(d)). TER dynamics in the presence of lysine was not significantly different from that in the presence of MDA alone although there was a positive trend for increased electric impedance of endothelial cells in the presence of lysine. NAC addition induced a sharp decrease in TER followed by a slow recovery toward the control TER values by the end of the experiment. Carnosine or lysine added at 2 mM protected EA.hy926 cells from deleterious effects of MDA. Comparatively, 2 mM carnosine increased TER of the EA.hy926 cell monolayer above the TER values achieved in the presence of 2 mM lysine or in the untreated control.

**Figure 2:**
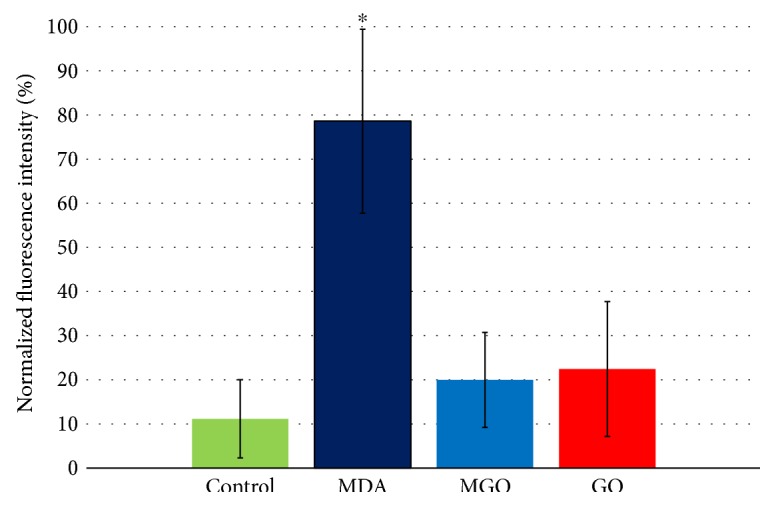
Effects of dicarbonyls on FITC-dextran diffusion through the EA.hy926 endothelial cell monolayer. Cells were challenged with 250 *μ*M MDA, GO, or MGO, and the amount of FITC-dextran diffused through the monolayer was measured 15 hours after its addition on top of the monolayer; this value was normalized by a maximum value of permeability attained in a particular experiment; data from three independent experiments were pooled. ^∗^*p* < 0.05 versus control, MGO, and GO (*n* = 10–12).

As the TER assay does not specifically measure permeability of an endothelial monolayer to macromolecules, we complemented the TER experiments by measuring FITC-dextran diffusion across the EA.hy926 cell monolayer.  Figure 2 shows that 15 hours after FITC-dextran addition, the highest fluorescent signal in the lower compartment of a diffusion chamber was achieved in the MDA-treated cells. The effects of either MGO or GO on FITC-dextran permeability across the EA.hy926 cell monolayer were not significant.

### 3.2. Differential Effects of Malondialdehyde versus Glyoxal/Methylglyoxal on Motility of Endothelial Cortical Cytoplasm

**Figure 3:**
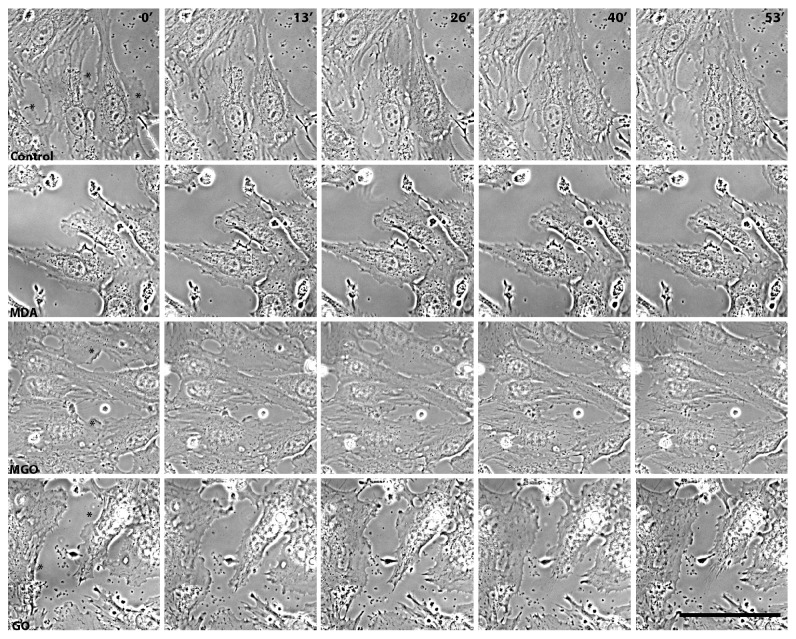
Time-matched frames from video recordings of lamellipodial motility in control and dicarbonyl-treated endothelial cells. Typical recordings for all conditions are shown. In 0′ frames, asterisks mark actively moving lamellipodia. Shown on the top row of images, timing in minutes applies to all rows. Phase contrast. Scale bar for all images: 50 *μ*m. Source movies of the presented frames are available as Supplementary files 1–4.

**Figure 4:**
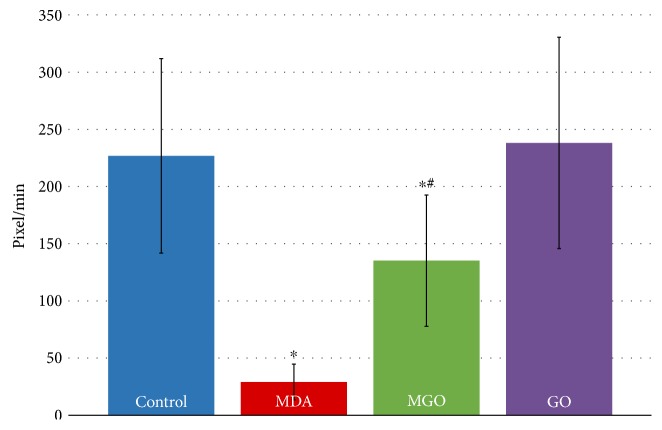
Speed of lamellipodia expansion/retraction in control and dicarbonyl-treated endothelial cells. The average speed of lamellipodia expansion or retraction was calculated from the video recordings of EA.hy926 cells as outlined in Materials and Methods (*n* = 10–12). ^∗^*p* < 0.01 versus control; ^#^*p* < 0.01 versus MDA.

As the barrier function of the endothelium depends on expansion of the cortical cytoplasm and formation of the adhesive contacts between the adjacent cells, we investigated the motility of the cortical cytoplasm after treatment of EA.hy926 cells with MDA, GO, or MGO. The cells were pretreated with dicarbonyls for 4 hours and monitored for an additional hour using the phase-contrast time-lapse microscopy.  Figure 3 shows the selected time-matched frames from these movies (the full movies are available as Supplementary files 1–4). In a subconfluent state, the control untreated cells demonstrate active formation and retraction of lamellipodia, through which cells establish stable or transient contacts with each other (Figure 4). Active lamellipodial dynamics was also observed in EA.hy926 cells treated with 250 *μ*M of GO or MGO, although in the latter case, the average speed of lamellipodial expansion/retraction decreased nearly 2-fold. In contrast, 250 *μ*M MDA substantially inhibited the lamellipodial motility. It markedly reduced frequency and amplitude of lamellipodium expansion and retraction, as well as the size and number of active lamellipodia. Mainly ruffling of the cortical cytoplasm was detected in MDA-treated cells (Supplementary file 2). Because of reduced lamellipodial activity, the MDA-treated cells stayed mostly retracted and did not establish contacts with each other. Still, the prolonged treatment of cells with 250 *μ*M MDA caused no cell death because in parallel to the ruffling, all cells exhibited active movements of intracellular organelles such as mitochondria and vesicles (Supplementary file 2). In addition, the standard Trypan blue exclusion viability test yielded similar results in the control and MDA-treated cells, 87% and 85%, respectively.

### 3.3. Differential Effects of MDA versus GO/MGO on Endothelial Cytoskeleton

**Figure 5:**
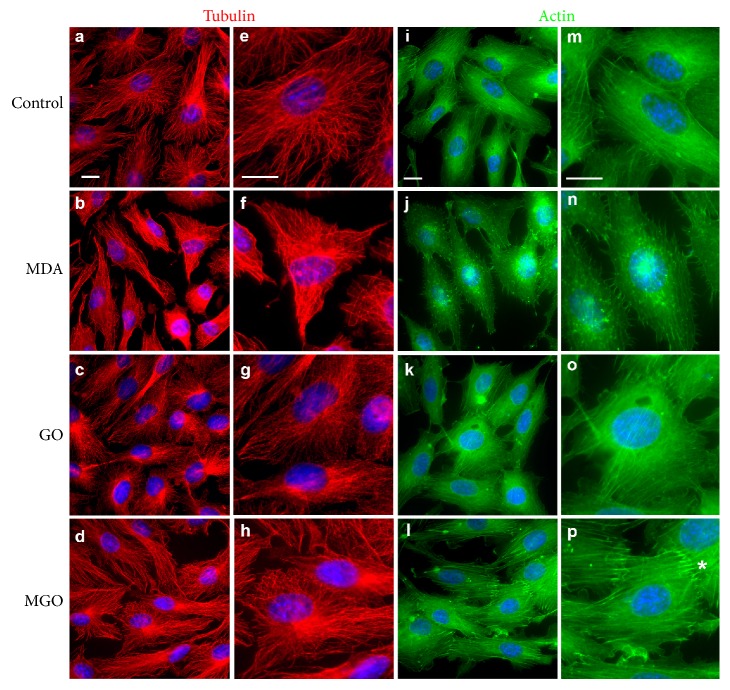
Effects of dicarbonyls on the organization of microtubules and filamentous actin in endothelial cells. General overview of tubulin or F-actin staining is shown in the left columns while an enlarged view of a corresponding image is given in the right columns for a better detail. ∗ indicates intense F-actin staining at focal contacts. Legends are near panels; colors are artificial. Scale bars: 10 *μ*m.

Cellular shape and motile reactions, including endothelial permeability, depend on the dynamic organization of the cytoskeleton. Therefore, we visualized two major cytoskeletal subsystems of EA.hy926 cells, the microtubules and microfilaments, using anti-tubulin antibodies and F-actin-specific reagent phalloidin, respectively. The panels in Figure 5 demonstrate the distribution of microtubules and microfilaments in the subconfluent EA.hy926 cells that were treated with vehicle (control cells) or 250 *μ*M of either MDA, GO, or MGO. In control cells, the microtubules were organized in a radial fashion and extended well toward the cell periphery reaching at a close distance the microtubules of the neighboring cells (Figures 5(a) and 5(e)). Similar arrangement of microtubules was observed in the cells treated with either GO or MGO. In the GO-treated cells, the microtubular network appears more faint and punctate, but still well spread (Figures 5(c) and 5(g)). In contrast, the majority of the MDA-treated cells lost their radial polarity of microtubules (Figures 5(b) and 5(f)). Their microtubules were distributed chaotically; many of them curved at the cell periphery. Thick bundles of microtubules were observed in some cells, while in other cells, the network collapsed to the perinuclear region.

In control endothelial cells, actin cytoskeleton was represented by the fine actin bundles that extended in parallel throughout the cell body and by the less structured perinuclear actin and nonprominent cortical actin that outlined lamellipodia (Figures 5(i) and 5(m)). The control cells established contacts with each other, either complete or partial with the unclosed gaps of variable size (Figure 5(m)). The cells treated with 250 *μ*M of GO or MGO demonstrated filamentous actin distribution similar to the control cells (Figures 5(k), 5(o), 5(l), and 5(p)). A distinct feature of cells exposed to 250 *μ*M MGO was augmentation of the focal contacts located at the ends of the actin bundles and manifested by more intense F-actin staining (indicated by an asterisk in Figure 5(p)). These structures were often observed in contact regions of the adjacent cells. In contrast, the actin bundles and perinuclear actin were largely reduced in the MDA-treated cells, whereas a large number of F-actin-positive bright dots and aggregates were observed (Figures 5(j) and 5(n)). Many cells exhibited the needle-like projections all around their periphery that were either free extending or contacting the neighbor cells. The MDA-treated cells demonstrated only few tight contacts of the type observed in the control cells, and such contacts usually spanned a short distance.

### 3.4. Differential Protein Modification in Endothelial Cells by MDA and MGO

**Figure 6:**
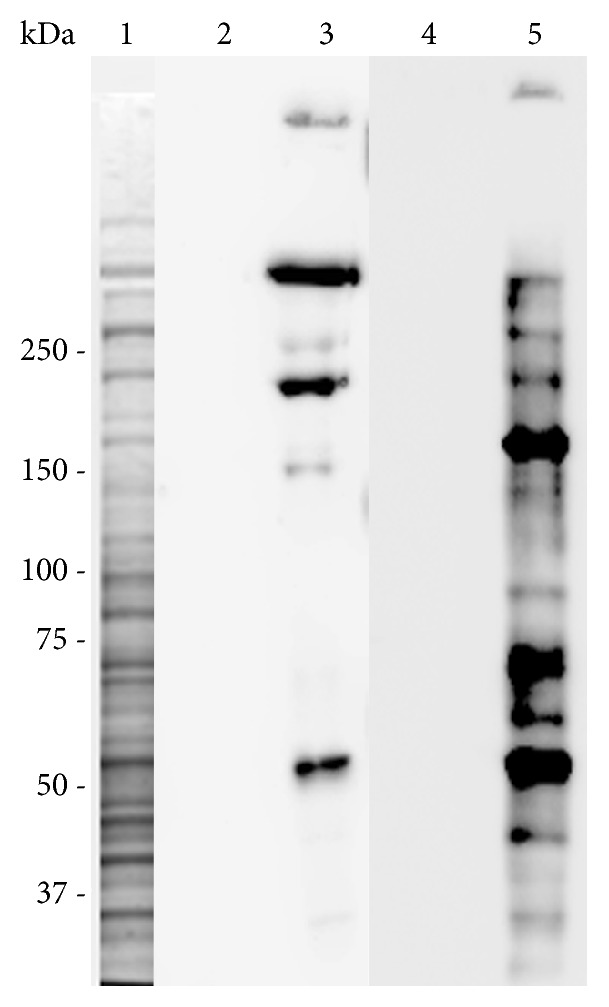
MDA and MGO modify different proteins in EA.hy926 cells. Western blotting of total EA.hy926 lysates after treatment of the cells with MDA or MGO was performed using the moAb against MDA- and MGO-labelled proteins (see Materials and Methods for details). 1: the Coomassie R-250-stained gel resolving the total EA.hy926 cell lysate; 2 and 3: Western blot of the control (2) or MDA-treated cells (3) with 3G4 moAb, which recognizes the MDA-labelled proteins; 4 and 5: Western blot of the control (4) or MGO-treated cells (5) with 6D8 moAb, which recognizes MGO-labelled proteins. Position of the molecular weight markers (Precision Plus Protein Dual Color-prestained MW standards, Bio-Rad) is shown on the left. Protein bands in all lanes are aligned based on mobility of MW standards.

We used in-house produced monoclonal antibodies that recognize MDA- or MGO-modified proteins in immunoblot lysates of EA.hy926 endothelial cells treated with 250 *μ*M of either MDA or MGO. As shown in Figure 6, both antibodies immunostained multiple protein bands on the total protein transfer of dicarbonyl-treated cells. The molecular weight distribution of the major immunostained protein bands in MDA- and MGO-treated cells was different but partially overlapping. The antibody to MDA-modified proteins intensely labeled the protein bands with the apparent molecular weights of around 50 kDa, 200 kDa, and above 250 kDa. The antibody to MGO-modified proteins revealed three major bands at 50 kDa, between 50 kDa and 75 kDa, and close to 150 kDa molecular weight markers. Alignment of Western blots with the Coomassie-stained gels of total EA.hy926 lysates revealed that the major MDA- and MGO-immunostained protein bands often corresponded to the major Coomassie-stained bands encompassing abundant cellular proteins (see Figure 6).

## 4. Discussion

Patients with atherosclerosis or diabetes patients with obesity present increased levels of MDA, MGO, and GO in blood plasma and tissues. These substances cause cellular damage through covalent modification and cross-linking of proteins and nucleic acids. In particular, they lead to dysfunction of the vascular endothelium including increased permeability to macromolecules.

Recent estimates of disease-related dicarbonyl levels using HPLC and mass spectrometry provide the values for GO and MGO concentrations in human plasma of 0.4 *μ*M and 0.2 *μ*M, respectively [22]. In type 2 diabetes patients, these values are about 1.5-fold higher. In the whole blood, the GO and MGO concentrations are 4-fold and 14-fold higher, respectively, than those in plasma [22, 23] indicating a higher cellular accumulation of these dicarbonyls. MDA concentration in human plasma falls in the range of 6–14 *μ*M [24], which exceeds GO and MGO plasma levels by an order of magnitude. Measurements of MDA content in the aortic tissue of rats with streptozotocin-induced diabetes yield the value of 2.18 ± 0.31 nmol per mg of soluble protein [25]. Assuming that soluble proteins mainly come from cells and approximately account for 30% of total cellular mass and that cells comprise about 20–30% volume in the arterial tissue [26], one can estimate the molar content of MDA being about 200 *μ*M in diabetic rat aorta and 2.5-fold less in normal aortic tissue [25]. An extensive search of the available literature failed to reveal any data on the MDA content in human diabetic tissues, and we assume that it is approximately the same as in the rat diabetes model. Hence, similar to MGO/GO distribution between plasma and cells, our calculations return by an order of magnitude the higher content of MDA in tissue than in plasma supporting the range of MDA concentrations used in this study.

Here, we compared the potency of MDA, MGO, and GO to compromise the endothelial barrier. A standard endothelial cell model based on the human EA.hy926 endothelial cell monolayer was chosen for experiments in order to avoid variability frequently associated with the primary endothelial cells. We found that at 150 *μ*M and above, MDA caused a profound increase in endothelial monolayer permeability whereas neither MGO nor GO had an effect. According to previous reports, the latter dicarbonyls increase endothelial permeability in vitro when used at concentrations of 600 *μ*M and 1–3 mM, respectively [9–11]. Noteworthy, the effect of MDA on endothelial permeability is irreversible in contrast to the reversible barrier attenuation induced by thrombin, a powerful edemagenic agent. Such behavior of endothelial cells is consistent with non-receptor-mediated randomized protein modification produced by the aldehyde, which contrasts the PAR1 receptor-mediated mechanism of thrombin. Thus, based on the side-by-side comparison, MDA was found considerably more damaging to the endothelial barrier as compared to either MGO or GO. In conjunction with the higher levels of MDA than MGO or GO in disease, this PUFA-derived dicarbonyl appears to surpass the glucose-derived dicarbonyls in its capacity to alter endothelial permeability in vascular pathologies associated with oxidative stress.

The natural dipeptide carnosine was successfully used to neutralize MDA toxicity toward the brain endothelial cells in vitro [27]; however, the authors measured other cellular parameters than permeability. It is well established that carbonyls react with free amino groups in molecules to form a Schiff base. Thus, a variety of primary amines could serve as MDA chemical quenchers. Carnosine has a free amino group at the *β*-alanine residue. Along with carnosine, we checked whether amino acid lysine, which possesses two amino groups, could protect the endothelium from the MDA attack. In addition, we tested NAC in the same TER assay as this compound is considered to attenuate oxidative stress and pathologic dicarbonyl accumulation. We compared the ability of these substances added at a concentration of 500 *μ*M to counteract action of 200 *μ*M MDA and revealed higher protective capacity of carnosine over lysine. At 2 mM concentration, both substances were protective; however, TER values were still higher in the presence of carnosine. Perhaps, the histidine residue of carnosine facilitates the Schiff base formation/stabilization, thus providing better reaction conditions than those in the case of the lysine-MDA interaction. In addition, endogenous concentrations of carnosine (2–20 mM) are in excess of those of lysine (90–150 *μ*M) and stay in a protective range against tissue levels of MDA. NAC also protected the endothelial barrier from MDA. However, the effect of NAC developed slowly and initiated with a steep decrease in TER like in the case of thrombin. These properties disfavor the use of NAC as a direct counter against MDA substance.

Endothelial permeability measured by TER and FITC-dextran diffusion is a paracellular-type permeability that depends on contacts between the adjacent endothelial cells within the monolayer [28]. In order to establish the intercellular contacts, endothelial cells spread toward each other extending lamellipodia. Using time-lapse microscopy, we found that MDA-treated endothelial cells fail to maintain normal lamellipodial activity and were limited to minor ruffling and blebbing of the cortical cytoplasm. These results are consistent with those of the permeability experiments, in which endothelial cells were in a denser monolayer but still could not maintain its integrity in the presence of MDA. In contrast, MGO-treated cells that also had somewhat reduced lamellipodial dynamics demonstrated no signs of barrier compromise. Apparently, the alterations produced by MGO were rather mild, and lamellipodial motility and adhesiveness of these cells remained sufficient to maintain integrity of the monolayer.

MDA modifies various cellular proteins including those involved in lamellipodial motility and intercellular adhesion, such as actin and tubulin isoforms, and those involved in the energy metabolism [29, 30]. Our fluorescent microscopy results corroborate this concept. We document the significant alterations in microtubule and microfilament networks in MDA-treated cells, which is consistent with its suppression of cortical cytoplasm motility, the loss of cellular contacts, and increased permeability to macromolecules. Obviously, in MDA-treated cells, the fenestrated contacts could not support the tight endothelial barrier unless spaces between them are filled by extending the cortical cytoplasm, the process inhibited in these cells.

Although it did not affect the endothelial barrier in our experiments, MGO did modify endothelial proteins as revealed by immunostaining of endothelial proteins on Western blots using the antibody against MGO-labelled proteins. Because of the lack of appropriate commercial antibodies, we were unable to visualize the GO-modified proteins in endothelial cells. However, based on the positive protein labelling by MGO, we assume that GO modified proteins in endothelial cells equally well, still without a detrimental effect on permeability, lamellipodial motility, and cytoskeleton.

In addition to different functional effects of MDA and MGO on the endothelium, the profiles of dicarbonyl-labelled protein bands in endothelial cells are mainly distinct. This could be attributed to chemical differences between these substances. MGO is a zero-length cross-linker whereas MDA is a longer molecule in which carbonyl groups are interspaced by an additional carbon atom. This allows MDA to reach more distantly located target groups in proteins that are not accessible to MGO. As a result, the effects of MDA versus MGO/GO on molecular dynamics of endothelial proteins might be quite different. Further support to this suggestion comes from our TER experiments using glutaraldehyde, a dialdehyde which carbonyl groups are separated by three carbon atoms instead of one in MDA. In the presence of glutaraldehyde, endothelial permeability increased faster than that in the presence of MDA.

Overall, we view the potential mechanism of MDA-induced endothelial hyperpermeability as a consequence of chemical modifications by this dicarbonyl of endothelial proteins involved in the maintenance of monolayer integrity. MDA efficiently forms cross-links within and between proteins and alters internal mobility of polypeptide chains, their interactions, and their functional properties. As a result, such protein-mediated cellular activities as lamellipodial motility and intercellular adhesion are compromised, and endothelial cells fail to establish the tight barrier. We suggest that protein cross-linking is more detrimental to these processes than just amino acid modifications that produce a mild phenotype.

## 5. Conclusions

Based on a side-by-side comparison, we identified MDA as a more aggressive dicarbonyl than either GO or MGO with regard to endothelial barrier dysfunction. Exceeding endogenous levels of MDA over GO and MGO and further accumulation of MDA during oxidative stress call for the development of therapies focused on the reduction of oxidative stress in general and MDA levels, in particular, in order to prevent sustained increases in vascular permeability and associated complications in obese diabetic patients and patients with atherosclerosis.

## Supplementary Material

Supplementary file 1. Control cells. Time-lapse phase-contrast movie of lamellopodial activity in control EA.hy926 endothelial cells. Supplementary file 2. MDA-treated cells. Time-lapse phase-contrast movie of lamellopodial activity in MDA-treated EA.hy926 endothelial cells. Supplementary file 3. MGO-treated cells. Time-lapse phase-contrast movie of lamellopodial activity in MGO-treated EA.hy926 endothelial cells. Supplementary file 4. GO-treated cells. Time-lapse phase-contrast movie of lamellopodial activity in GO-treated EA.hy926 endothelial cells.

## References

[1] Jenkins A. J., Hill M. A., Rowley K. G., Holtzman J. L. (2007). Diabetes and oxidant stress. *Atherosclerosis and Oxidant Stress*.

[2] Lankin V., Konovalova G., Tikhaze A., Shumaev K., Kumskova E., Viigimaa M. (2014). The initiation of free radical peroxidation of low-density lipoproteins by glucose and its metabolite methylglyoxal: a common molecular mechanism of vascular wall injure in atherosclerosis and diabetes. *Molecular and Cellular Biochemistry*.

[3] Lykkesfeldt J. (2007). Malondialdehyde as biomarker of oxidative damage to lipids caused by smoking. *Clinica Chimica Acta*.

[4] Lange J. N., Wood K. D., Knight J., Assimos D. G., Holmes R. P. (2012). Glyoxal formation and its role in endogenous oxalate synthesis. *Advances in Urology*.

[5] Simm A. (2013). Protein glycation during aging and in cardiovascular disease. *Journal of Proteomics*.

[6] Ayala A., Munoz M. F., Arguelles S. (2014). Lipid peroxidation: production, metabolism, and signaling mechanisms of malondialdehyde and 4-hydroxy-2-nonenal. *Oxidative Medicine and Cellular Longevity*.

[7] Lankin V. Z., Konovalova G. G., Tikhaze A. K. (2016). Aldehyde inhibition of antioxidant enzymes in the blood of diabetic patients. *Journal of Diabetes*.

[8] Kumskova E. M., Antonova O. A., Balashov S. A., Tikhaze A. K., Melkumyants A. M., Lankin V. Z. (2014). Malonyldialdehyde and glyoxal act differently on low-density lipoproteins and endotheliocytes. *Molecular and Cellular Biochemistry*.

[9] Sliman S. M., Eubank T. D., Kotha S. R. (2010). Hyperglycemic oxoaldehyde, glyoxal, causes barrier dysfunction, cytoskeletal alterations, and inhibition of angiogenesis in vascular endothelial cells: aminoguanidine protection. *Molecular and Cellular Biochemistry*.

[10] Toth A. E., Toth A., Walter F. R. (2014). Compounds blocking methylglyoxal-induced protein modification and brain endothelial injury. *Archives of Medical Research*.

[11] Toth A. E., Walter F. R., Bocsik A. (2014). Edaravone protects against methylglyoxal-induced barrier damage in human brain endothelial cells. *PLoS One*.

[12] Chaplen F. W., Fahl W. E., Cameron D. C. (1996). Method for determination of free intracellular and extracellular methylglyoxal in animal cells grown in culture. *Analytical Biochemistry*.

[13] Requena J. R., Fu M. X., Ahmed M. U. (1997). Quantification of malondialdehyde and 4-hydroxynonenal adducts to lysine residues in native and oxidized human low-density lipoprotein. *The Biochemical Journal*.

[14] Edgell C. J., McDonald C. C., Graham J. B. (1983). Permanent cell line expressing human factor VIII-related antigen established by hybridization. *Proceedings of the National Academy of Sciences of the United States of America*.

[15] Khapchaev A., Samsonov M. V., Kazakova O. A. (2012). Suppression of vascular endothelium hyperpermeability by cell-permeating peptide inhibitors of myosin light chain kinase. *Biofizika*.

[16] Marchenko A. V., Sidorova M. V., Sekridova A. V. (2009). Penetrating peptide inhibitor of the myosin light chain kinase suppresses hyperpermeability of vascular endothelium. *Rossiĭskii Fiziologicheskiĭ Zhurnal Imeni I.M. Sechenova*.

[17] Lankin V. Z., Afanasieva O. I., Konovalova G. G. (2011). Modification of lipoprotein(a) by natural dicarbonyls induced their following free radical peroxidation. *Doklady Biochemistry and Biophysics*.

[18] Yanushevskaya E. V., Valentinova N. V., Medvedeva N. V., Morozkin A. D., Vlasik T. N. (1999). Low density lipoprotein heterogeneity tested by monoclonal antibodies. *Angiology and Vascular Surgery*.

[19] Kudryashov D. S., Stepanova O. V., Vilitkevich E. L. (2004). Myosin light chain kinase (210 kDa) is a potential cytoskeleton integrator through its unique N-terminal domain. *Experimental Cell Research*.

[20] Laemmli U. K. (1970). Cleavage of structural proteins during the assembly of the head of bacteriophage T4. *Nature*.

[21] Towbin H., Staehelin T., Gordon J. (1979). Electrophoretic transfer of proteins from polyacrylamide gels to nitrocellulose sheets: procedure and some applications. *Proceedings of the National Academy of Sciences of the United States of America*.

[22] Scheijen J. L., Schalkwijk C. G. (2014). Quantification of glyoxal, methylglyoxal and 3-deoxyglucosone in blood and plasma by ultra performance liquid chromatography tandem mass spectrometry: evaluation of blood specimen. *Clinical Chemistry and Laboratory Medicine*.

[23] Fleming T., Cuny J., Nawroth G. (2012). Is diabetes an acquired disorder of reactive glucose metabolites and their intermediates?. *Diabetologia*.

[24] Moselhy H. F., Reid R. G., Yousef S., Boyle S. P. (2013). A specific, accurate, and sensitive measure of total plasma malondialdehyde by HPLC. *Journal of Lipid Research*.

[25] Cho W. C., Chung W. S., Lee S. K., Leung A. W., Cheng C. H., Yue K. K. (2006). Ginsenoside Re of *Panax ginseng* possesses significant antioxidant and antihyperlipidemic efficacies in streptozotocin-induced diabetic rats. *European Journal of Pharmacology*.

[26] Vermeulen E. G., Niessen H. W., Bogels M., Stehouwer C. D., Rauwerda J. A., van Hinsbergh V. W. (2001). Decreased smooth muscle cell/extracellular matrix ratio of media of femoral artery in patients with atherosclerosis and hyperhomocysteinemia. *Arteriosclerosis, Thrombosis, and Vascular Biology*.

[27] Hipkiss A. R., Preston J. E., Himswoth D. T., Worthington V. C., Abbot N. J. (1997). Protective effects of carnosine against malondialdehyde-induced toxicity towards cultured rat brain endothelial cells. *Neuroscience Letters*.

[28] Mehta D., Malik A. B. (2006). Signaling mechanisms regulating endothelial permeability. *Physiological Reviews*.

[29] Pamplona R. (2011). Advanced lipoxidation end-products. *Chemico-Biological Interactions*.

[30] Zarkovic N., Cipak A., Jaganjac M., Borovic S., Zarkovic K. (2013). Pathophysiological relevance of aldehydic protein modifications. *Journal of Proteomics*.

